# Distorting Nature

**Published:** 1883-03

**Authors:** 


### DISTORTING NATURE.

"An American missionary, Miss Norwood, of Swatow, has lately described how the size of the foot is reduced in Chinese women. The binding of the foot is not begun till the child has learned to walk and do various things. The bandages are specially manufactured, and are about two inches wide and two yards long for the first year, five yards long for subsequent years. The end of the strip is laid on the inside of the foot at the instep, then carried over the toes, under the foot, and around the heel, the toes being thus drawn toward and over the sole, while a bulge is produced on the instep and a deep indentation in the sole. The indentation, it is considered, should measure about an inch and a half from the part of the foot that rests on the ground up to the instep. Successive layers of bandages are made till the strip is all used, and the end is then sewn tightly down. The foot is so squeezed upward that in walking only the ball of the great toe touches the ground. Large quantities of powdered alum are used to prevent ulceration and lessen the offensive odor. After remaining a month in this condition, the foot is soaked in hot water for some time; then the bandage is carefully unwound, much dead cuticle coming off with it. Ulcers and sores are often found on the foot; frequently, too, a large piece of flesh sloughs off the sole, and one or two toes may even drop off, in which case the woman feels afterward repaid by having smaller and more delicate feet. Each time the bandage is taken off the foot is kneaded, to make the joints more flexible, and is then bound up again as quickly as possible with a fresh bandage, which is drawn up more tightly. During the first year the pain is so intense that the sufferer can do nothing, and for about two years the foot aches continually, and is the seat of a pain which resembles the pricking of sharp needles.

"With continued rigorous binding the foot in two years becomes dead and ceases to ache, and the whole leg, from the knee down, becomes so shrunken as to be little more than skin and bone. When once formed, the 'golden lily,' as the Chinese lady calls her delicate little foot, can never more recover its original shape."

COMMENTS.—The Chinese woman simply distorts her feet, while her *civilized* American sister cramps heart, lungs, and digestive organs *by her tight lacing!* The "heathen Chinee" suffers pain only for a few years, but our fair ladies suffer for many years their self-inflicted torture! Headache, cold extremities, poor digestion, hence poor complexion (not to mention their *monthly* ills), are a few effects of this pernicious fashion.

We may well ask, which is the more heathenish, to distort one's feet or one's heart, lungs, and digestive organs? The poor Chinese women have the laugh on you, my fair friends! Have physicians no duties to perform here? Can they not abate this sin?

